# Supplementary material for: Risk Factors for Mortality of Hospitalized Adult Patients with COVID-19 Pneumonia: A Two-Year Cohort Study in a Private Tertiary Care Center in Mexico
Source: Int J Environ Res Public Health. 2023 Mar 2;20(5):4450. doi: 10.3390/ijerph20054450 (PMC10001871; doi:10.3390/ijerph20054450)

Supplementary Figure S1. MULBSTA, Charlson and NEWS scales comparisons between survivors and non-survivors.

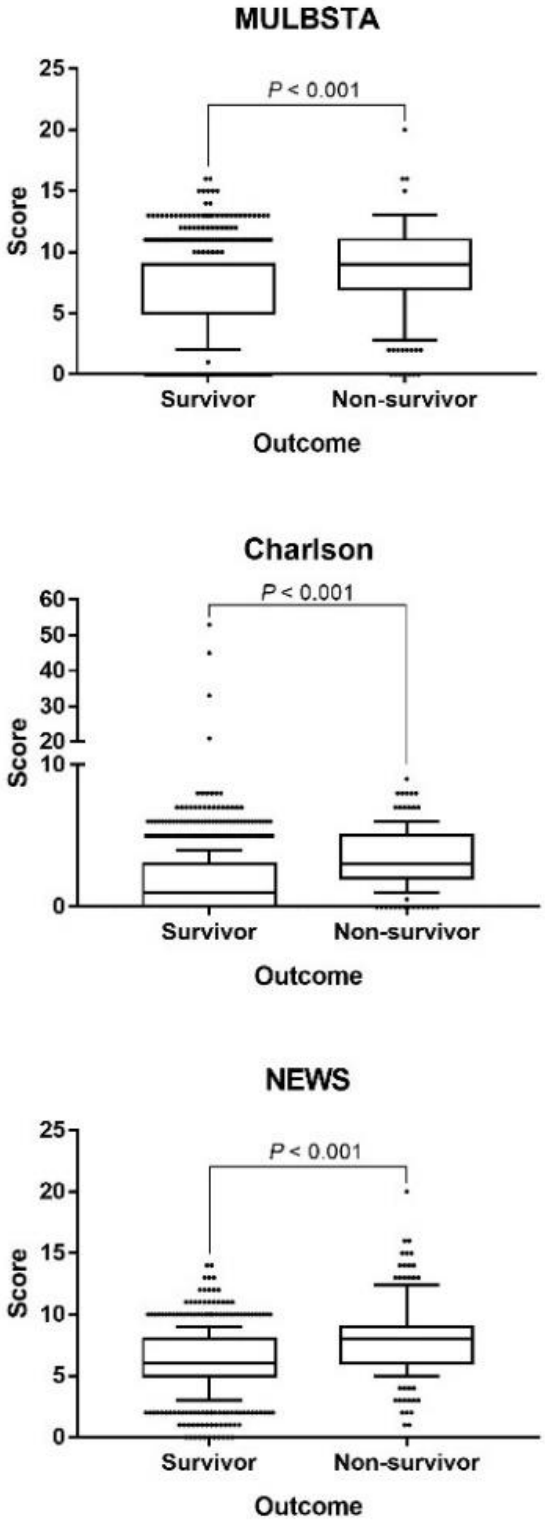

Supplement: Supplementary file 1 [file ijerph-20-04450-s001.zip › Supplementary Figure S1.pdf]
