# Supplementary material for: Risk Factors for Mortality of Hospitalized Adult Patients with COVID-19 Pneumonia: A Two-Year Cohort Study in a Private Tertiary Care Center in Mexico
Source: Int J Environ Res Public Health. 2023 Mar 2;20(5):4450. doi: 10.3390/ijerph20054450 (PMC10001871; doi:10.3390/ijerph20054450)
Supplement: Supplementary file 1 [file ijerph-20-04450-s001.zip › Supplementary Table S1.pdf]

**Supplementary Table S1. Causes of death**

| *Cause* | *n* | *%* |
|---|---|---|
| Multi-organic failure | 70 | 42.4 |
| ARDS | 56 | 33.9 |
| Septic shock | 18 | 10.9 |
| Unstable bradycardia | 6 | 3.6 |
| Myocardial infarction | 4 | 2.4 |
| Pulmonary embolism | 5 | 3 |
| Hypovolemic shock | 4 | 2.4 |
| Unstable supraventricular tachycardia | 1 | 0.6 |
| Stroke | 1 | 0.6 |
