# Supplementary material for: Risk Factors for Mortality of Hospitalized Adult Patients with COVID-19 Pneumonia: A Two-Year Cohort Study in a Private Tertiary Care Center in Mexico
Source: Int J Environ Res Public Health. 2023 Mar 2;20(5):4450. doi: 10.3390/ijerph20054450 (PMC10001871; doi:10.3390/ijerph20054450)
Supplement: Supplementary file 1 [file ijerph-20-04450-s001.zip › Supplementary Table S2.pdf]

Supplementary Table S2. Vaccination

Variable	Total n (%)	Survivor n (%)	Non-survivor n (%)	*P* value*
Vaccinated (n = 166)	12.6%	17.2%	15.3%	0.618&
Astra Zeneca (n = 72)	5.7%#	41.8%	55.0%	
Sinovac (n = 20)	1.6%#	11.6%	15.0%	
Pfizer (n = 52)	4.1%#	33.6%	15.0%	
CanSino (n = 7)	0.6%#	2.7%	2.7%	
J&J (n = 9)	0.7%#	15.0%	15.0%	
Moderna (n = 6)	0.5%#	6.2%	6.2%	

&Survivor vs Non-survivor; #percentages are respect vaccinated patients; *Chi-square[2]
