# Supplementary material for: Risk Factors for Mortality of Hospitalized Adult Patients with COVID-19 Pneumonia: A Two-Year Cohort Study in a Private Tertiary Care Center in Mexico
Source: Int J Environ Res Public Health. 2023 Mar 2;20(5):4450. doi: 10.3390/ijerph20054450 (PMC10001871; doi:10.3390/ijerph20054450)
Supplement: Supplementary file 1 [file ijerph-20-04450-s001.zip › Supplementary Table S3.pdf]

Supplementary Table S3. Chi-squared tests including observed and expected values

Variable	Total<br>n (%)	Survivor<br>n (%)<br>*expected value*	Non-survivor<br>n (%)<br>*expected value*	*P* value[1]
Sex (Male/Female), n	845/413	741/352<br>*734.2/358.8*	104/61<br>*110.8//54.2*	0.248*
BMI classification, %				
Underweight	10 (0.8%)	9 (0.8%)<br>*8.7*	1 (0.6%)<br>*1.3*	
Normal BMI	240 (19.1%)	199 (18.4%)<br>*208.5*	41 (24.8%)<br>*31.5*	
Overweight	486 (38.6%)	423 (38.3%)<br>*422.3*	63 (38.2%)<br>*63.7*	0.399*
Obesity grade I	322 (25.6%)	285 (26.1%)<br>*279.8*	37 (22.4%)<br>*42.2*	
Obesity grade II	114 (9.1%)	103 (9.3%)<br>*99.0*	11 (6.7%)<br>*15.0*	
Obesity grade III	86 (6.8%)	74 (7.0%)<br>*74.7*	12 (7.3%)<br>*11.3*	
Hypertension (%)	427 (33.9%)	346 (31.7%)<br>*371.0*	81 (49.1%)<br>*56.0*	<0.001*
Diabetes (%)	270 (21.5%)	216 (19.8%)<br>*234.6*	54 (32.7%)<br>*35.4*	<0.001*
COPD (%)	34 (2.7%)	22 (2.0%)<br>*29.5*	12 (7.3%)<br>*4.5*	0.001*
Asma (%)	27 (2.1%)	25 (2.3%)<br>*23.5*	2 (1.2%)<br>*3.5*	0.422*
Immunosuppression (%)	56 (4.5%)	42 (3.8%)<br>*48.7*	14 (8.5%)<br>*7.3*	0.010*
Cancer (%)	60 (4.8%)	42 (3.8%)<br>*52.1*	18 (10.9%)<br>*7.9*	<0.001*
HIV (%)	2 (0.2%)	2 (0.2%)<br>*1.7*	0 (0%)<br>*0.3*	>0.999*
Previous stroke (%)	16 (1.3%)	11 (1.0%)<br>*13.9*	5 (3.0%)<br>*2.1*	>0.999*
CKD (%)	49 (3.9%)	39 (3.6%)<br>*42.6*	10 (6.1%)<br>*6.4*	0.021*
Organ transplant recipient (%)	16 (1.3%)	14 (1.3%)<br>*13.9*	2 (1.2%)<br>*2.1*	>0.999*
Chronic liver disease (%)	9 (0.7%)	8 (0.7%)<br>*7.8*	1 (0.6%)<br>*1.2*	>0.999*
ACEI/ARA (%)	283 (22.5%)	234 (21.4%)<br>*245.9*	49 (29.7%)<br>*37.1*	0.021*
Previous myocardial infarction (%)	35 (2.8%)	23 (2.1%)<br>*30.4*	12 (7.3%)<br>*4.6*	0.001*
Depression (%)	17 (1.4%)	15 (1.4%)	2 (1.2%)	0.830*

		14.8	*2.2*	
Smoker (%)				
Never	808 (64.2%)	715 (65.4%)	93 (56.4%)	
		702.0	*106.0*	
Unknown	301 (23.9%)	257 (23.5%)	44 (26.7%)	0.071*
		261.5	*39.5*	
Currently	65 (5.2%)	54 (4.9%)	11 (6.7%)	
		56.5	*8.5*	
Former	84 (6.7%)	67 (6.1%)	17 (10.3%)	
		73.0	*11.0*	
Alcohol use (%)				
Never	805 (64.0%)	700 (64.0%)	105 (63.6%)	
		699.4	*105.6*	
Unknown	330 (26.2%)	279 (25.5%)	51 (30.9%)	0.114*
		286.7	*43.3*	
Currently	109 (8.7%)	102 (9.3%)	7 (4.2%)	
		94.7	*14.3*	
Former	14 (1.1%)	12 (1.1%)	2 (1.2%)	
		12.2	*1.8*	
COVID-19 pneumonia on imaging (%)	1,133 (90.1%)	981 (90.2)	152 (92.1)	0.480*
		983.8	*149.2*	
NIH severity scale (%)				
Critical	64 (5.1%)	26 (2.4%)	38 (23.0%)	
		55.6	*8.4*	
Moderate	90 (7.2%)	85 (7.8%)	5 (3.0%)	<0.001*
		78.2	*11.8*	
Severe	1,104 (87.8%)	982 (89.8%)	122 (73.9%)	
		959.2	*144.8*	
qSOFA (%) n				
0	354 (28.1%)	329 (30.1%)	25 (15.2%)	
		307.6	*46.4*	
1	816 (64.9%)	714 (65.3%)	102 (61.8%)	<0.001*
		709.9	*107.0*	
2	71 (5.6%)	47 (4.3%)	24 (14.5%)	
		61.7	*9.3*	
3	17 (1.4%)	3 (0.3%)	14 (8.5%)	
		14.8	*2.2*	
Fever (%) n	756 (60.1%)	658 (60.2%)	98 (59.4%)	0.865*
AVPU score				
Alert	1,196 (95.1%)	1,066 (97.5%)	130 (78.8%)	
		1039.1	*156.9*	
Voice	30 (2.4%)	17 (1.6%)	13 (7.9%)	<0.001*
		26.1	*3.9*	
Pain	7 (0.6%)	1 (0.1%)	6 (3.6%)	
		6.1	*0.9*	
Unresponsive	25 (2.0%)	9 (0.8%)	16 (9.7%)	

		21.7	*3.3*	
Cough (%)	838 (66.6%)	726 (66.4%)<br>*728.1*	112 (67.9%)<br>*109.9*	0.724*
Headache (%)	538 (42.8%)	478 (43.7%)<br>*467.4*	60 (36.4%)<br>*70.6*	0.077*
Dyspnea (%)	1,041 (82.8%)	892 (81.6%)<br>*904.5*	149 (90.3%)<br>*136.5*	0.008*
Diarrhea (%)	252 (20.0%)	224 (20.5%)<br>*218.9*	28 (17.0%)<br>*33.1*	0.300*
Chest pain (%)	224 (17.8%)	193 (17.7%)<br>*194.6*	31 (18.8%)<br>*29.4*	0.743*
Chills (%)	309 (24.6%)	275 (25.7%)<br>*268.5*	34 (16.4%)<br>*40.5*	0.210*
Odynophagia (%)	308 (24.5%)	281 (37.4)<br>*267.6*	27 (29.7%)<br>*40.4*	0.011*
Myalgias (%)	458 (36.4%)	409 (37.4%)<br>*397.9*	49 (29.7%)<br>*60.1*	0.057*
Arthralgias (%)	406 (32.3%)	357 (32.7%)<br>*352.7*	49 (29.7%)<br>*53.3*	0.476*
Malaise (%)	880 (70.0%)	769 (70.4%)<br>*764.6*	111 (67.3%)<br>*115.4*	0.466*
Rhinorrhea (%)	151 (12.0%)	135 (12.4%)<br>*131.2*	16 (9.7%)<br>*19.8*	0.370*
Vomiting (%)	93 (7.4%)	83 (7.6%)<br>*80.8*	10 (6.1%)<br>*12.2*	0.529*
Abdominal pain (%)	77 (6.1%)	64 (5.9%)<br>*66.9*	13 (7.9%)<br>*10.1*	0.382*
Conjunctivitis (%)	22 (1.7%)	20 (1.8%)<br>*19.1*	2 (1.2%)<br>*2.9*	0.757*
Cyanosis (%)	101 (8.0%)	71 (6.5%)<br>*87.8*	30 (18.2%)<br>*13.2*	<0.001*
Anosmia (%)	145 (11.5%)	135 (12.4%)<br>*126.0*	10 (6.1%)<br>*19.0*	0.018*
Dysgeusia (%)	129 (10.3%)	118 (10.8%)<br>*112.1*	11 (6.7%)<br>*16.9*	0.129*
Glasgow Coma Scale <15 (%)	77 (6.1%)	43 (3.9%)<br>*66.9*	34 (20.6)<br>*10.1*	<0.001*

[1]Comparisons are between survivors and non-survivors; *chi-square test with Exact Fisher's test if apply.
